# Anorectal Melanoma: A Case Report

**DOI:** 10.7759/cureus.48835

**Published:** 2023-11-15

**Authors:** Papa Amoako-Teming, Pouya Rostami, Pawan Mehta, Imran Saeed

**Affiliations:** 1 Department of Surgery, St. Peter's University Hospital, New Brunswick, USA; 2 Department of Surgery, St. George's University School of Medicine, Great River, USA; 3 Department of Surgery, St. Peter’s University Hospital - Rutgers Robert Wood Johnson School of Medicine, New Brunswick, USA

**Keywords:** melanoma skin cancer, malignant melanoma initial presentation, mucosal malignant melanoma, rectal malignant melanoma, anorectal melanoma

## Abstract

Anorectal mucosal melanoma (AMM) is an infrequent and highly aggressive form of mucosal melanoma. Its rarity makes it challenging to clinically diagnose, and its initial symptoms are typically nonspecific such as rectal/anal bleeding (the most common symptom), anal pain, or the presence of an anal mass. The prognosis for this condition is generally poor, and its incidence appears to be increasing each year. AMMs often go undetected and/or are already metastasized at the time of diagnosis. We present a case report of a patient who initially presented with nonspecific symptoms of anemia and blood per rectum, and was later found to have stage IV melanoma of the anorectal region. There is a notable scarcity of literature on this disease, resulting in a lack of a comprehensive understanding of its nature. Most available information consists of isolated case reports rather than comprehensive studies. Although surgical resection remains the primary treatment approach, the majority of patients (over 80%) will die due to distant metastasis within five years after undergoing surgery. The five-year survival rate for anorectal melanoma is estimated to be between 6% and 22%.

## Introduction

Skin cancer is the most prevalent type of cancer, with invasive melanoma accounting for approximately 1% of all skin cancers. Although most melanomas manifest on the skin (i.e., cutaneous melanomas), there are also mucosal melanomas that develop in the mucous membranes of the body, including the sinuses, nasal passages, oral cavity, vagina, anus, and rectum [1]. Mucosal melanomas account for approximately 1.4% of all melanomas, with roughly 50% originating in the head and neck region. A greater proportion of the remaining cases occur in the anorectal area and female genitalia, whereas a smaller percentage affects the esophagus, gallbladder, bowel, conjunctiva, and urethra [2]. Anorectal mucosal melanoma (AMM) accounts for approximately 0.4%-1.6% of all malignant melanomas and 4% of anal malignancies [2,3].

The incidence of melanoma has been steadily increasing over the past few decades; according to the Centers for Disease Control and Prevention, the incidence rate in the United States from 2012 to 2016 was 21.8 cases per 100,000 individuals. The highest incidence was observed among non-Hispanic Caucasian males, whereas the lowest incidence was reported among individuals of African descent. In 2023, the American Cancer Society estimates that approximately 186,680 new cases of melanoma will be diagnosed in the United States, with 97,610 of those being invasive melanoma (58,120 in men and 39,490 in women). The society also predicts that 7,990 people (5,420 men and 2,570 women) will die from the disease [4].

Melanoma develops when melanocytes undergo mutations, leading to their uncontrolled proliferation and transformation into cancerous cells. These mutations can be acquired sporadically (most common) or inherited through germline mutations. Exposure to ultraviolet (UV) light, primarily from sunlight, is identified as the primary risk factor for developing melanoma, according to the American Cancer Society [5]. Other risk factors include a family history of the disease, the presence of atypical moles, male sex, fair skin, freckling, light hair, immunocompromised individuals, aging, and conditions such as xeroderma pigmentosum. Race is also a significant predictor of melanoma development [2].

Treatment for melanoma varies depending on the stage of the disease (i.e., stages I-IV) and is most effective in the early stages when it is confined to the epidermal skin layer. With an improved understanding of the disease’s pathogenesis, progression, and immunology, treatment modalities have been rapidly evolving. Options include immunotherapy, targeted therapy, radiation therapy, chemotherapy, and surgical resection, with surgery being the preferred first-line therapy.

The purpose of this case report is to further bring into light a highly morbid type of melanoma in the hopes of promoting earlier detection and consequently better prognosis.

## Case presentation

The patient was a 79-year-old Caucasian male with a medical history of hypertension, hyperlipidemia, coronary artery disease (status post-stent placement), obesity (body mass index = 31.1 kg/m^2^), and gastroesophageal reflux disease. He initially presented with anemia and blood per rectum while wiping. During a recent hospital admission, his hemoglobin level was found to be 5.4 g/dL, and he received five units of packed red blood cells and two units of fresh frozen plasma. On physical examination, external hemorrhoids were observed, along with an ulcerated hypertrophic papilla-shaped lesion. The mass felt soft upon palpation. Initial investigations included an upper endoscopy, which did not identify a bleeding source, and a colonoscopy, which revealed a hard, ulcerated lesion 1 cm in length in the anal canal adjacent to the hemorrhoids. The patient had no history of inflammatory bowel disease or any prior anorectal procedures.

A surgical consult was obtained, and the patient underwent transanal excision of the anal canal lesion. The patient was placed in a prone position, and intravenous sedation was administered. The perianal area was infiltrated with 20 cc of bupivacaine for local anesthesia. The lesion in the anal canal was identified, and a full-thickness excision was performed using the Harmonic scalpel. The resulting defect was closed with 3-0 vicryl sutures. The incision was irrigated, hemostasis was achieved, and an additional 15 cc of 0.25% bupivacaine was infiltrated for further anesthesia. The patient remained stable throughout the procedure and tolerated it well.

Pathology results revealed invasive malignant melanoma of the anal canal, with the specimen measuring 2.4 cm in its longest diameter. The tumor exhibited ulceration and lymphovascular invasion, and the distal margin was positive. The tumor involved the anal mucosa, extended into the columnar colonic mucosa, and had a mitotic rate of 15/10 high-power fields. Immunohistochemical marker studies, including pankeratin, S100, Melan A, HMB45, and Ki67, supported the diagnosis.

A fluorodeoxyglucose (FDG) positron emission tomography scan was performed, which showed no appreciable FDG uptake within the rectum. However, evaluation of pelvic structures was limited due to a streak artifact from the patient’s right hip prosthesis. The scan revealed an FDG-avid left upper lobe pulmonary nodule indicative of metastatic disease, as well as focal FDG-avid skin thickening within the right parietal scalp, raising concern for malignancy.

At this stage, the patient’s disease was classified as stage IV with lung involvement. Further surgical interventions, such as wide local excision (WLE), would have required excision of the sphincters but would not have altered the patient’s prognosis. Therefore, the patient was referred to an oncologist for further management of the disease.

## Discussion

AMMs often face delayed diagnosis due to their nonspecific initial presentation. A study revealed that 41% of such tumors had already spread regionally, and 22% had metastasized distantly by the time they were detected [6]. The lungs, liver, brain, and bone are the typical sites of metastasis [7].

The most common initial symptom of AMM is painless rectal bleeding. Due to the rarity of this neoplasm, other conditions, such as hemorrhoids, polyps, and even squamous cell carcinoma are usually considered first and ruled out. In larger tumors with deeper infiltration, symptoms may become more noticeable, including bleeding, the presence of an anal mass, pain, constipation, and weight loss [8].

Although melanomas are typically pigmented, if a dark-colored mass is observed at the anal verge, it can be mistaken for a thrombosed hemorrhoid. Additionally, AMMs can present as amelanotic melanomas, lacking pigmentation and requiring histopathological evaluation for diagnosis [9].

Melanoma originates from melanocytes, the pigment-producing cells in the skin. Melanocytes are found in the basal layer of the epidermis and are responsible for producing melanin, the pigment that gives color to the skin and provides protection against UV radiation, acting as a natural “sunscreen.” In mucosal membranes, melanocytes also play a role in antimicrobial defense and immune responses [10].

The exact pathogenesis of AMM remains limited and not fully understood [10]. Several theories exist regarding its development. Some propose a relation to oxidative stress in the anal region and/or immunosuppression. Others suggest derivation from Schwannian neuroblastic cells or cells of the amine-precursor uptake and decarboxylation system of the gut [11]. The KIT receptor tyrosine kinase has also been implicated in the development of malignant melanoma, including AMM. Loss-of-function mutations, as well as activating mutations in genes such as KIT, BRAF, and NRAS, have been associated with melanoma development [2].

Due to the rarity of AMM, there are not sufficient randomized trials and standardized treatment plans for the disease. However, surgical interventions are commonly employed [10]. WLE or abdominoperineal resection (APR) are the preferred surgical approaches, with similar outcomes. Considering the morbidity associated with APR, WLE is generally recommended [12]. Intermediate-thickness mucosal melanoma may require sentinel lymph node biopsy [13].

Various adjuvant therapies have been utilized in the literature. Alfa-interferon immunotherapy has shown increased survival and reduced recurrence rates in node-positive patients. Other treatments, such as 117-Caesium brachytherapy and chemotherapy regimens containing dacarbazine, vincristine, and nimustine hydrochloride, have been used with varying success rates [14]. Treatment decisions should consider the patient’s quality of life and comorbidities, especially for metastatic AMM.

Immunotherapy has shown promising outcomes in recent years [10]. Agents such as ipilimumab (anti-CTLA-4 monoclonal antibody), nivolumab, and pembrolizumab (anti-PD1 monoclonal antibodies) have been used to target T-cell-mediated antitumor immune responses [10,15]. Imatinib mesylate has shown encouraging results in patients with KIT-mutated rectal melanoma, and combination therapies (e.g., nivolumab + ipilimumab) have proven effective in advanced melanoma [16,17]. Sunitinib has been utilized to achieve complete remission in patients with KIT-mutated melanoma [18]. Radiation therapy has not been extensively studied but has been used for hemostasis in certain cases [19].

Early diagnosis and staging are crucial factors influencing the prognosis of AMM [20]. Ottaviano et al. have proposed a flowchart for the diagnosis, staging, and treatment of AMM [20]. Unfortunately, the prognosis for AMM is generally poor, with an estimated five-year survival rate of approximately 20% regardless of the treatment modality used. The main objective of surgical intervention is to improve the patient’s quality of life, as many individuals with AMM already have extensive metastasis at the time of diagnosis. Perineural invasion, as identified on histopathology, is the most significant factor affecting prognosis and the likelihood of recurrence [2].

## Conclusions

Anorectal mucosal melanoma is an extremely aggressive disease characterized by its rarity and nonspecific initial presentation, making its early detection challenging. Currently, surgical resection is the preferred treatment option, but there is a lack of comprehensive research on the disease itself. Our objective was to contribute to the existing body of case studies of AMM, raise awareness about its high morbidity and mortality rates, and aid in the development of guidelines for its management.

The diagnosis of skin cancer, including AMM, heavily relies on patient detection. However, this approach often leads to missed lesions, especially considering the obscurity of AMM. To address this issue, we propose implementing full-body skin surveys as part of annual physical examinations. Given that skin cancers account for approximately one-third of reported cancers worldwide, incorporating regular skin examinations can help with early detection. Additionally, we recommend annual anoscopies with particular attention to the dentate line due to the aggressive nature of melanomas in the anorectal region.
